# Teleworking in times of a pandemic: An applied study of industrial companies

**DOI:** 10.3389/fpsyg.2022.1061529

**Published:** 2022-11-17

**Authors:** Laura Becerra-Astudillo, Belén Vargas-Díaz, Carlos Molina, Jorge Serrano-Malebrán, Fernando Garzón-Lasso

**Affiliations:** ^1^Department of Administration, Catholic University of the North, Antofagasta, Chile; ^2^Business and Organization Research Group, Business School, EAFIT University, Medellín, Colombia

**Keywords:** telework, trust, job satisfaction, work-family, pandemic (COVID-19)

## Abstract

The purpose of this study is to determine the effect of teleworking on self-reported job satisfaction and workers’ productivity in the context of the COVID-19 pandemic. A survey was administered to 331 teleworkers belonging to industrial companies, whose data were analyzed with a PLS-SEM structural equation model. The results indicate that communication with coworkers, time spent teleworking, and workplace suitability positively affect self-reported productivity, while trust on the part of supervisors and workplace suitability positively affect job satisfaction. On the other hand, work-family conflict negatively affects job satisfaction and self-reported productivity, whereas communication with coworkers, support from supervisor and time spent teleworking have no significant effect on job satisfaction. This study provides relevant information for industrial organizations to improve the job-satisfaction and productivity in large scaled teleworking contexts, as should have been implemented during the mandatory preventive isolation due to the health crisis related to the transmission of SARs-CoV-2.

## Introduction

The health crisis resulting from COVID-19 has brought unprecedented changes in people’s daily lives and one of the consequences has been the sudden increase in teleworking, as organizations have been forced to take on this modality of work as a preventive measure of contagion (Belzunegui and Erro, 2020; Matamala, 2020; Davidescu et al., 2021; Mahmood et al., 2021; Petcu et al., 2021; Sousa-Uva et al., 2021; T̨ălnar-Naghi, 2021). The International Labor Organization (ILO) estimates that before the pandemic only 2.9% of employees worldwide teleworked permanently from home (Berg et al., 2020). However, nowadays, many countries have adopted this modality (International Labour Organization [ILO], 2020). This is evident in developed countries, such as the USA, since before the pandemic only 15% of workers used this modality of work and currently about 50% of the USA workforce is teleworking (Brynjolfsson et al., 2020). On the other hand, telework also experienced an important growth in Latin American countries, a clear example of this being Chile. According to the statistics, since the health crisis began, 54% of workers have been working from home (GeoVictoria, 2020).

The potential for teleworking varies globally, because not all countries have the same infrastructure available (Berg et al., 2020). Likewise, not all countries have regulations that control this work modality. Among the countries with regulations, collective agreements and/or public policies, the USA, France, Belgium, Italy, Spain, Portugal, Brazil, Colombia, and Argentina stand out (Álvarez, 2018; Organización Internacional del Trabajo [OIT], 2021). Likewise, Chile has joined these countries, since, because of the contingency, the authorities were pressured to enact the “Remote Work and Telework Law” in March 2020, whose project had been in process since 2018 (Diario Financiero, 2020).

While teleworking has certain advantages, it also brings disadvantages when not implemented correctly (Courtney, 2020; Davidescu et al., 2021). Within the positive effects, results such as higher job satisfaction, better job performance, among others, have been found (Nakrošienë et al., 2019; Schall, 2019; Toscano and Zappalà, 2020). On the other hand, negative effects include professional isolation, increased work-family conflict, decreased productivity, and job dissatisfaction (Milton et al., 2016; Delanoeije et al., 2019; Nakrošienë et al., 2019). Currently, the effect of pandemic telework on self-reported worker job satisfaction and productivity is unknown because previous research has studied the impact of teleworking under what might be termed normal conditions, other than the current COVID-19 pandemic scenario. Therefore, this study aims to determine the effect of factors associated with telework on self-reported job satisfaction and the productivity of workers belonging to industrial companies in the context of the COVID-19 pandemic. The methodological design of this research has a quantitative approach, where independent and dependent variables are included. These were obtained from the work of Nakrošienë et al. (2019), where three variables of telework were identified: “support by the supervisor,” “time dedicated to teleworking,” and “suitability in the workplace.” For the purposes of this study, the variables “communication with co-workers” and “work-family conflict” were added to the model. On the other hand, the population is centered on the workers of the different companies that belong to the Antofagasta Industrial Association (AIA), where the collection of information was carried out utilizing surveys to then carry out a structural equation model.

This study provides a relevant contribution to the actual literature review. First, while most of the studies were conducted on developed countries, it was carryout in Chile, a country leading economic growth in Latin America. Second, although the region has lower telework indicators than developed countries, it shows clear signs of strengthening infrastructure and government laws that better regulate this labor relationship and, therefore, are paving the way for more hybrid work. Third, the research offers a better understanding of the effect of telework on workers’ self-reported job satisfaction and productivity during a pandemic, especially in Antofagasta-Chile, where studies like this are incipient. Finally, it also has implications for managers, as it helps them rethink how to implement telework, especially considering that once the pandemic is over, many organizations have reported that they will use a blended or hybrid system. On the other hand, at the social level, it allows governments to understand the critical points to work on regarding regulations and/or policies that protect workers.

This study begins with a review of the literature and the hypotheses proposed, followed by the research methodology. Subsequently, the results obtained are provided, and finally, the conclusions of the study are presented.

## Literature review

### Evolution of the concept of telework

The concept of telework has evolved throughout history. Nilles (1975) first defined the concept of “Telecommunications” as a network composed of computer and telecommunication components, which allows workers in large organizations to work in offices near their homes, to replace long-distance travel to a central office. Subsequently, the term Telecommunications was scaled up to “Telework” to include all work-related activities enabled by information and communitation technology (ICT) outside the employer’s infrastructure (Nilles, 1988). A decade later, Daniels et al. (2001) established that telework deviates from standard working conditions in flexible practice arrangements. Later the concept of telework is established and defined in broader terms, such as “Flexible Work” and “Mobile Work.” Shockley and Allen (2007) postulated “Flexible Work” as work options that allow work activities to be performed outside the traditional boundaries, in terms of time and place of a standard workday. Likewise, Martínez et al. (2006) posited that “Mobile Work” is an action where work-related activities are performed in different locations using telecommunications technology. Further on, Eurofound and the International Labour Office (2017) and Eurofound (2022) stated that telework is a type of work and/or service performed remotely, at a distance, and online through a computer and telematic technologies.

The evolution of the concept of telework has been possible, largely thanks to the advancement of telecommunications technology and information society (Karácsony, 2021), because this has generated the concept changes, being more feasible and profitable since the way of working has been transformed (Messenger and Gschwind, 2016). Therefore, within the literature reviewed, there is no single definition for telework. Finally, in this research, the definition that will be used is the following: Telework is a work practice that involves members of an organization who perform their work activities away from the central office, usually from home, using ICT to interact with others and execute work tasks. This definition is based on different conceptualizations proposed by various authors (Nilles, 1988; Shockley and Allen, 2007).

### Theorical foundations of telework factors and their relationship to self-reported job satisfaction and productivity

From the work of Nakrošienë et al. (2019), three telework variables were identified, which are “supervisor support and trust,” “time spent teleworking,” and “workplace suitability.” For this study, two variables are added to the model. First, “communication with coworkers” is added since telework brings important changes, mainly in workers’ communication (Peiró and Soler, 2020). Second, the variable “work-family conflict” is included because several studies have related this conflict as being one of the main factors that impact the job satisfaction of teleworkers (Delanoeije et al., 2019; Schall, 2019; Sousa-Uva et al., 2021). For these reasons the above variables are considered relevant to the study and interesting to analyze. Each of these is described below.

#### Communication with co-workers

Communication with coworkers when coworkers are teleworking can be positively and negatively related to job satisfaction and workers’ self-reported productivity (Fay and Kline, 2011; Milton et al., 2016). From a positive perspective, Fay and Kline (2011) argue that communication with coworkers is positively related to teleworkers’ job satisfaction. On the other hand, some authors posit that communication has a positive impact on worker productivity (Muñoz, 2012; Haapakangas et al., 2018). Therefore, proper communication with coworkers increases the productivity of teleworkers. This is because a good communication flow allows employees to work more efficiently, since “bureaucratic communication” is minimized, which helps to streamline work processes and also contributes to clearer planning of tasks so that the time spent by each person is much more productive (Muñoz, 2012; Randstad, 2017). On the contrary, Milton et al. (2016) and Mahmood et al. (2021) argue that workers may feel a sense of isolation, loss of contact, and relationship with coworkers when working from home for longer periods. This is mainly because when working remotely, communication is different, i.e., face-to-face communication is reduced (Svein, 2009). According to Vasquez (2015), communication with coworkers and supervisors is directly related to job satisfaction, so it is understood that a reduction in communication can cause dissatisfaction in workers. From the literature, the following hypotheses are proposed:

**H1:** Communication with coworkers when people are teleworking has a positive relationship with job satisfaction.

**H2:** Communication with coworkers when people are teleworking has a positive relationship with self-reported productivity.

#### Supervisor support and trust

Supervisor support and trust with teleworkers are important factors directly related to overall employee satisfaction (Nakrošienë et al., 2019). Al Omar et al. (2011) and Dang and Hong (2020) argue a positive association between supervisor trust and employee job satisfaction. Therefore, if organizations seek to increase satisfaction by implementing telework, managers should make an effort to improve supervisor support with workers (Bae et al., 2019). Furthermore, this is supported by Houghton et al. (2018), who argue that the development of support and trust by supervisors with teleworkers leads to benefits in working conditions and employee satisfaction. This is because trust contributes to the development of positive attitudes at work, such as competence, sincerity, integrity, respect, and credibility, which results in higher job satisfaction (Tan and Lim, 2009). The following hypotheses are put forward:

**H3:** Trust on the part of the supervisor when people are teleworking has a positive relationship with job satisfaction.

**H4:** The support from the supervisor when people are teleworking has a positive relationship with job satisfaction.

#### Time spent teleworking

Telework hours increase productivity in the workplace, as long as these are appropriate. On the other hand, when telework hours are too long, teleworkers decrease their job satisfaction and productivity (Asociación de Iniciativas Sociales [AISS], 2016; Dockery and Bawa, 2018; Kazekami, 2020; Mahmood et al., 2021). On the contrary, some authors argue that a factor that positively influences productivity is the longer time spent teleworking. This is because people who work from home have more time than people who work from the office since they do not have to spend their time commuting from home to the office, in addition to teleworkers determining for themselves the working hours (Icare, 2020; Thorstensson, 2020). Similarly, this is supported by Acosta (2018), who indicates that a factor that positively affects productivity is that workers can devote more time to telework because they do not have the distractions of the office, perceive a reduction in travel time, have greater self-management of time, and, in addition, they need not waste their time on activities with less importance. The following hypotheses are presented:

**H5:** Time spent teleworking has a negative relationship with job satisfaction.

**H6:** Time spent teleworking has a positive relationship with self-reported productivity.

#### Suitability in the workplace

An ideal workplace setting is important when implementing teleworking, as the suitability of the location where teleworking takes place strengthens satisfaction and increases productivity (Nakrošienë et al., 2019; Organización Internacional del Trabajo [OIT], 2020). This is further supported by Ansong and Boateng (2018), who argue that factors related to the use of technology, such as devices and compatible technology, including the Internet, make people telework better. Because having physical comfort in the workspace meets the needs of workers, it provides them with better concentration and comfort, such as appropriate furniture to perform the work. In addition, a suitable environment supports the tasks performed, which in turn allows for fostering employee satisfaction (Budie et al., 2019). Also, having adequate space and basic materials, such as a desk, chair, and printer, among others, have become necessary elements for people to telework more productively (Pinola, 2020). The following hypotheses are proposed:

**H7:** Workplace appropriateness when people are teleworking has a positive relationship with job satisfaction.

**H8:** Workplace appropriateness when people are teleworking has a positive relationship with self-reported productivity.

#### Work-family conflict

Work-family conflict is defined as “*a form of role conflict in which the pressures of the work and family roles are mutually incompatible in some respect. That is, participation in the work/family role is difficult because of participation in the family/work role*” (Greenhaus and Beutell, 1985, p. 77). According to the literature reviewed, this conflict when people are teleworking can be related to job satisfaction and productivity in both negative and positive ways. From a positive perspective, Schall (2019) and Davidescu et al. (2021), propose that teleworking can lead to a decrease in work-family conflict because there are fewer interruptions in working with the family, which leads to greater satisfaction on the part of the worker with his/her job. From a negative perspective, Delanoeije et al. (2019) propose that teleworking has detrimental aspects for the worker, since the boundaries become more permeable and easy to cross, which can generate conflicts within the household (Gajendran and Harrison, 2007). This is due to the possibility of the working day being extended and productive and domestic work being interspersed (Bosch et al., 2020). Teleworking has also increased gender differences regarding the division of activities at home (Villarreal, 2020; Lara-Pulido and Martinez-Cruz, 2022). This leads to negative consequences for both the company and the workers, producing low productivity on workers (Kossek and Ozeki, 1999), which would also affect their levels of job satisfaction (Kossek and Ozeki, 1998; Hernández, 2019). The following hypotheses are proposed:

**H9:** Work-family conflict when people are teleworking has a negative relationship with job satisfaction.

**H10:** Work-family conflict when people are teleworking has a negative relationship with self-reported productivity.

The proposed model and its hypotheses are shown in Supplementary Figure 1 below.

## Methodology

### Sample

The data was collected through a survey between the months of August and November 2020 in Chile. The survey was aimed at workers belonging to member companies of the AIA, who at some point during the pandemic teleworked or were still teleworking. The AIA brings together more than 240 mining companies and suppliers. The organization provided us with contacts with companies and workers. The surveys were done in personal interviews by professional surveyors. To eliminate possible ambiguities in the questionnaire, a pilot test was applied to 70 teleworkers. After making minor changes to the instrument, face-to-face surveys were carried out. The exclusion of invalid surveys provided a final sample of 331 teleworkers. A total of 51.1% of the participants were women, and 69.9% worked in a large company. The majority of the respondents were between 25 and 43 years of age, 62.8%. A total of 30.2% said they would like to continue teleworking after the pandemic, 22.7% said they would not, and 47.1% would like to continue teleworking only sometimes. As for the intensity of teleworking, 55% of the respondents teleworked full-time, while 45% teleworked part-time. Finally, 57.1% of the participants were offered teleworking, 38.7% were forced to do so, and only 4.2% requested it.

### Measurement scales

A survey based on constructs from the previous literature was applied. All the items were evaluated on a 5-point Likert scale, where one is “strongly disagree,” and five is “strongly agree,” except for the sociodemographic variables and others related to telework characteristics. Supplementary Table 1 presents each construct’s name, the variable type, Cronbach’s Alpha, the author, and the year corresponding to each construct.

### Analysis techniques

Structural equation modeling (SEM), specifically partial least squares (PLS), was proposed to test the reliability, validity, and proposed hypotheses. The PLS-SEM allows the evaluation of both causal relationships between indicators/items and the causal relationships of latent constructs (Gudergan et al., 2008). The procedures suggested in the previous literature (Fornell and Larcker, 1981; Wright et al., 2012; Henseler et al., 2016) were used to evaluate the measurements and the structural model.

## Results

### Construct validity and reliability

For the evaluation of the measurement model, the criteria of reliability, convergent validity and discriminant validity of all the multiple-item scales were evaluated. Supplementary Table 2 shows Cronbach’s Alpha coefficient (CA), the composite reliability (CR), Dijkstra-Hernseler’s indicator (rhoa_A), in addition to the loadings of each item, with which the reliability of the model was evaluated. For the proposed variables, the individual reliability of each item is ensured through loadings of more than 0.7 in its corresponding latent variable. Second, the reliability of the constructs was analyzed using Cronbach’s alpha criteria (values between 0.827 and 0.965) and the composite reliability (values between 0.928 and 0.974). In all the cases, the indicators are greater than 0.7. Additionally, convergent validity is ensured by analyzing the average extracted variance. In the analysis, values are between 0.743 and 0.904. All the indicators offer levels above the 0.5 score proposed in the literature. Discriminant validity was established according to the Fornell-Larcker criterion (Fornell and Larcker, 1981), in which the square root values of the AVE found on the diagonal are observed to be greater than the correlations between the constructs (Supplementary Table 3). Similarly, the Heterotrait-Monotrait Ratio criterion (Voorhees et al., 2016) was considered, which presented adequate values, lower than 0.9, in all the relationships (Henseler et al., 2016). Therefore, the results obtained reveal that there is indeed discriminant validity (Supplementary Table 4).

### Structural model

The evaluation of the structural model is carried out to test the proposed hypotheses. This is done through the Standardized Root Mean Residual (SRMR), the Path coefficients, and the *R*^2^ values. The SRMR was used to measure the overall model fit. This yielded a value of 0.065, indicating a good fit for the proposed model (Prasarnphanich and Wagner, 2009). On the other hand, path loadings and p-values were calculated to analyze the relationships described in the hypotheses, calculated with the bootstrapping technique of 5,000 subsamples. Supplementary Figure 2 shows the results of the proposed model. In addition, the *R*^2^ values that were verified to evaluate the predictive capacity of the structural model are shown. Also, Supplementary Table 5 shows the values of the Path coefficients and their *p*-values.

## Discussion

A short time after the COVID-19 pandemic was declared by the WHO as a public health emergency of global concern, relevant data on the impact of telework on workers’ perceptions and behaviors were not abundant. This phenomenon represents a key challenge for academics and practitioners. To address this limitation, we have successfully achieved the objective of this study to determine the effect of telework on the self-reported job satisfaction and productivity of workers belonging to member companies of the AIA in the context of the COVID-19 pandemic.

To fulfill the objective of this study, we first proposed a theoretical model that includes latent constructs related to telework and its effects on job satisfaction and self-reported productivity. Second, we evaluated the model with a structural equation approach. The results show that the proposed model is a convenient tool to explain job satisfaction and self-reported productivity in the context of telework during the COVID-19 pandemic. This is demonstrated by the validity and reliability of the scales, the variance explained, and the fit of the structural model. We must emphasize the parsimony of the model, the *R*^2^ values show that a reduced number of variables allows explaining a high percentage of job satisfaction and self-reported productivity.

The results show that communication with coworkers has a positive effect on self-reported productivity (H2). These results are similar to those obtained in a study on work from home carried out in Romania during the COVID-19 pandemic, whose authors, based on qualitative research, proposed that team spirit, which includes interaction and integration between colleagues, influences employee productivity in teleworking contexts (Săvescu et al., 2022). This relationship is consistent with the previous literature’s findings that communication has a positive impact on worker productivity (Muñoz, 2012; Haapakangas et al., 2018). This may be because good communication minimizes “bureaucratic communication,” which in turn allows streamlining of work processes so that the time spent by each person will be much more productive (Muñoz, 2012; Randstad, 2017). In fact, good coordination with colleagues was denominated as best practice by Doberstein and Charbonneau (2022) to “ensure that deadlines are met and projects are finished on time” (p. 16) in the context of telework in the Pandemic, according to the results obtained by them in a study developed in Canada.

Trust on the part of supervisors positively affects job satisfaction (H3). These results are consistent with what was proposed by Al Omar et al. (2011), who argue that there is a positive association between trust on the part of the supervisor and workers’ job satisfaction. This is because trust contributes to developing positive attitudes at work, which translates into higher job satisfaction (Tan and Lim, 2009). Time spent teleworking has a positive effect on self-reported productivity (H6), which coincides with those effects postulated by Thorstensson (2020), who points out that people who work from home have more time to devote to work than people who work from the office, since they do not have to spend their time commuting from home to the office, and they also have fewer distractions.

The data indicate that workplace suitability positively affects job satisfaction (H7), this relationship is consistent with that proposed by Nakrošienë et al. (2019) and Guayacán Rabelo et al. (2022) since the telework environment or the since the choice of a good workplace is relevant when implementing telework because it strengthens the satisfaction of collaborators (Budie et al., 2019). Workplace suitability has a positive effect on self-reported productivity (H8), being consistent with what was postulated by Nakrošienë et al. (2019) and Pinola (2020), who conclude that the suitability of the place where teleworking is performed and having good support resources, such as a good chair and printer, increase productivity. This is further supported by Sánchez (2007) and Thorstensson (2020), who argue that having efficient ICT, such as having access to fast internet, positively impacts productivity. Related to both results, in a research during the pandemic, Mihalca et al. (2021) found “that good physical conditions and adequate telework tools would be positively related to job performance and satisfaction with telework” (p. 631). Now, to guarantee a good place for teleworking and improve job satisfaction and productivity, organizations could adapt space in the teleworker’s home, or rent spaces for teleworking in a shared co-working office, which, according to Lara-Pulido and Martinez-Cruz (2022) is an attractive alternative for office workers in Mexico even before COVID-19, where employees were willing to pay due to the benefits in terms of time and cost that it brings.

Work-family conflict negatively affects job satisfaction (H9). This is in line with the literature reviewed, where it is argued that teleworking has detrimental aspects for the worker as boundaries become more permeable and easy to cross, which can generate conflicts within the household (Delanoeije et al., 2019). In addition, due to the pandemic, work and family must be forcibly reconciled, which generates an increase in conflict, decreasing workers’ job satisfaction (Kossek and Ozeki, 1998; Yaccar, 2020). Finally, work-family conflict negatively affects self-reported productivity (H10). This result is consistent with that proposed by Bosch et al. (2020), who postulate that when teleworking is not properly regulated or there are gaps in its regulation, the working day can be extended to such an extent that it conflicts with domestic tasks. This generates low productivity on the part of the workers and with it affects the organizational productivity (Kossek and Ozeki, 1999). Unfortunately, regulations worldwide are still deficient, as teleworkers must navigate unequal regulations, rules and work practices to fulfill their work activities, which increases the chances of having work-family conflicts that obviously could negatively affect their productivity levels (Dauplaise et al., 2020).

The results show that communication with coworkers has no significant effect on job satisfaction (H1). This is in contrast to what was proposed by Fay and Kline (2011), who points out that increased communication with coworkers can increase workers’ job satisfaction levels. This discrepancy may be explained by the pandemic since by carrying out work activities from home, other factors such as the conditions of the physical space and intrafamily relationships take on a greater relevance when monitoring workers’ satisfaction (Araque, 2020). The results indicate that support from supervisors does not have a significant effect on job satisfaction (H4). This result differs from the previous literature, where it is argued that support by the supervisor generates in workers a greater commitment to the organization, trust and job satisfaction because employees behave based on the treatment they receive (Bae et al., 2019). This discrepancy may occur due to the supervisor’s support not being as relevant when teleworkers have a greater sense of freedom, have less control and have more self-management, improving their motivation levels to meet their goals (Cervantes, 2005). Finally, the results reveal that time spent teleworking does not have a significant effect on job satisfaction (H5). This contradicts the previous literature. Kazekami (2020) indicates that when telework hours are too long, teleworkers decrease their job satisfaction. One of the possible arguments for what was found in this study is that, despite teleworkers having longer working hours, they also enjoy greater autonomy and a better quality of life (Lampert and Poblete, 2018). In addition, a shorter workday translates into less time to complete work-related tasks, which can cause teleworkers the opposite effect on job satisfaction, such as higher stress levels, fatigue, and work intensification.

## Conclusion

A short time after the COVID-19 pandemic was declared by WHO as a public health emergency of global concern, relevant data on the impact of telework on job satisfaction and self-reported productivity of workers was not abundant. To address this limitation, the objective of this study, to determine the impact of telework on the self-reported job satisfaction and productivity of workers belonging to member companies of the AIA in the context of the COVID-19 pandemic, has been successfully achieved.

The results of this research show how trust on the part of the supervisor contributes to increased job satisfaction of workers when they are teleworking. In addition, communication with coworkers allows teleworkers to be more productive. Similarly, when workers devote more time to telework, it impacts productivity positively. Also, the suitability of the location where telework is performed strengthens satisfaction and increases productivity. Conversely, working from home can increase levels of conflict at home, which generates a decrease in job satisfaction and worker productivity.

This study has certain implications for researchers since it provides a greater understanding of workers’ perception of telework and its relationship with job satisfaction and self-reported productivity in a pandemic scenario, specifically in the Second Region of Antofagasta. The study results are also of interest to managers since it allows them to rethink how to implement telework, even more so considering that once the pandemic ends, many organizations intend to maintain a face-to-face or hybrid telework system. Similarly, they must think about how to implement a telework system that allows them to reconcile work with family or generates a minimum impact. Finally, at the social level, it enables governments to understand the critical aspects that should be enhanced regarding regulations, agreements and/or public policies for the workforce to not feel unprotected.

On the other hand, this research has some limitations to be considered in future studies. First, the scope of the research was focused on similar organizations, mainly centering on the Mining-Industrial sector in the Second Region of Antofagasta-Chile. Second, the research is limited to studying only certain variables, such as communication with co-workers, trust and support from the supervisor, time spent teleworking, suitability in the workplace and work-family conflict. Third, the study is limited to measuring only self-reported productivity, i.e., teleworkers’ perceptions of their productivity. While subjective ratings are cost-effective and can be easily obtained, their reliability is often questioned, as there are many risks of cognitive biases (e.g., correspondence bias, personal relevance bias, egocentric bias, or social desirability effects).

Given the results obtained about a direct and positive relationship between the suitability of the location where telework and satisfaction and productivity, future research could study whether the best alternative to guarantee a good physical and technological space is in the teleworker home adapted by the company or in shared offices near the home of the teleworker. In addition to this, it is recommended that future studies consider more diverse economic sectors of telework. It is also suggested to study the impact of telework in companies from a different perspective, such as, for example, its effect on organizational culture and workers’ level of attachment. Finally, we suggest expanding the domain of some variables like Workspace Suitability with themes beyond space, lighting and ventilation, considering issues such as ergonomics.

## Data availability statement

The raw data supporting the conclusions of this article will be made available by the authors, without undue reservation.

## Ethics statement

The studies involving human participants were reviewed and approved by the Universidad Católica del Norte. The patients/participants provided their written informed consent to participate in this study.

## Author contributions

LB-A, BV-D, and CM: literature review and data. JS-M: methodology and results. FG-L: discussion and conclusion. All authors contributed to the article and approved the submitted version.
