# Supplementary material for: Teleworking in times of a pandemic: An applied study of industrial companies
Source: Front Psychol. 2022 Nov 17;13:1061529. doi: 10.3389/fpsyg.2022.1061529 (PMC9715348; doi:10.3389/fpsyg.2022.1061529)
Supplement: Supplementary file 1 [file Data_Sheet_1.docx]

Supplementary Material

## Supplementary Tables

Table 1. Constructs obtained from different instruments

| **Construct Name** | **Tiype of Variable** | **Cronbach's Alpha** | **Author** | **Year** |
| --- | --- | --- | --- | --- |
| **Communication with coworkers** | Independent | 0.96 | Hernández | 2019 |
| **Supervisor support** | Independent | 0.97 | Eisenberger | 1986 |
| **Confidence from supervisor** | Independent | 0.935 | Dietz & Hartog | 2006 |
| **Time spent teleworking** | Independent | 0.84 | Green | 2019 |
| **Workplace suitability** | Independent | 0.96 | Hernández | 2019 |
| **Work-family conflict** | Independent | 0.95 | Netemeyer | 1996 |
| **Job satisfaction** | Dependent | 0.911 | Brayfield & Rothe | 1951 |
| **Self-reported productivity** | Dependent | 0.84 | Meulen | 2010 |

Table 2. Reliability and validity of the variables

| ***Variable*** |  | ***ALL*** |
| --- | --- | --- |
| **Communication with coworkers** (CM) | All: AVE: 0.845 CR: 0.942 CA: 0.908 Rho_A: 0.909 |  |
|  | My relationship with my coworkers when I telework is adequate (CM 1) | 0.904 |
|  | I have communicated frequently with my coworkers since I teleworked (CM 2) | 0.914 |
|  | The information I receive from my coworkers is clear and useful when I telework (CM3) | 0.940 |
| **Confidence of the supervisor** (CPS) | All: AVE: 0.904 CR: 0.974 CA: 0.965 Rho_A: 0.968 |  |
|  | My supervisor sees me as a dedicated employee when I telecommute (CPS 1) | 0.950 |
|  | My supervisor believes that I perform adequately when I work at home (CPS 2) | 0.962 |
|  | When I work from home my supervisor considers me a reliable person (CPS 3) | 0.942 |
|  | My supervisor has full confidence in me when I telework (CPS 4) | 0.950 |
| **Supervisor support** (APS) | All: AVE: 0.845 CR: 0.928 CA: 0.897 Rho_A: 0.901 |  |
|  | My supervisor values my contribution to the well-being of the organization when I work from home (APS 1) | 0.838 |
|  | My supervisor really cares about my well-being when I telecommute (APS 2) | 0.905 |
|  | My supervisor cares about my overall satisfaction in teleworking (APS 3) | 0.872 |
|  | My supervisor takes pride in my accomplishments in the work I do at home (APS 4) | 0.881 |
| **Time spent teleworking** (TDT) | All: AVE: 0.743 CR: 0.945 CA: 0.931 Rho_A: 0.956 |  |
|  | I am sure that I telework more than the normal working hours (TDT 1) | 0.884 |
|  | It is necessary to telework more than the normal working hours to do my job (TDT 2) | 0.768 |
|  | When I telework, I have to work more than expected (TDT 3) | 0.912 |
|  | I continue to telework after the regular workday ends (TDT 4) | 0.861 |

|  | In general. I work more hours than usual when I telework (TDT 5) | 0.911 |
| --- | --- | --- |

|  | I spend more time on tasks related to telecommuting than when I work from the office (TDT 6) | 0.829 |
| --- | --- | --- |
| **Workplace suitability** (ILT) | All: AVE: 0.802 CR: 0.924 CA: 0.879 Rho_A: 0.910 |  |
|  | The space available to me for teleworking is adequate (ILT 1) | 0.910 |
|  | The lighting in the teleworking place is adequate (ILT 2) | 0.914 |
|  | Ventilation in the telework place is pleasant (ILT3) | 0.862 |
| **Work-family conflict** (CTF) | All: AVE: 0.816 CR: 0.957 CA: 0.943 Rho_A: 0.944 |  |
|  | The demands of telework interfere with my home and family life (CTF 1) | 0.922 |
|  | The amount of time teleworking requires makes it difficult for me to fulfill my family responsibilities (CTF 2) | 0.924 |
|  | Things I want to do at home don't get done because of the demands of my work from home (CTF 3) | 0.902 |
|  | Teleworking causes me stress, and this makes it difficult for me to fulfill my family obligations (CTF 4) | 0.884 |

Table 3. Discriminant validity, Fornell-Larcker criterion

|  | APS | CM | CPS | CTF | ILT | PA | SL | TDT |  |
| --- | --- | --- | --- | --- | --- | --- | --- | --- | --- |
| APS | **0.874** |  |  |  |  |  |  |  |  |
| CM | 0.563 | **0.919** |  |  |  |  |  |  |  |
| CPS | 0.743 | 0.677 | **0.951** |  |  |  |  |  |  |
| CTF | -0.159 | -0.264 | -0.096 | **0.903** |  |  |  |  |  |
| ILT | 0.431 | 0.399 | 0.371 | -0.225 | **0.896** |  |  |  |  |
| PA | 0.401 | 0.531 | 0.485 | -0.361 | 0.457 | **0.870** |  |  |  |
| SL | 0.394 | 0.433 | 0.394 | -0.509 | 0.384 | 0.740 | **0.823** |  |  |
| TDT | 0.067 | -0.048 | 0.136 | 0.636 | -0.107 | -0.090 | -0.291 | 0.862 |  |

Table 4. Discriminant validity, Heterotrait-Monotrait Ratio (HTMT) criterion.

|  | **APS** | **CM** | **CPS** | **CTF** | **ILT** | **PA** | **SL** | **TDT** |
| --- | --- | --- | --- | --- | --- | --- | --- | --- |
| **APS** |  |  |  |  |  |  |  |  |
| **CM** | **0.617** |  |  |  |  |  |  |  |
| **CPS** | 0.789 | **0.724** |  |  |  |  |  |  |
| **CTF** | 0.173 | 0.284 | **0.101** |  |  |  |  |  |
| **ILT** | 0.489 | 0.442 | 0.402 | **0.241** |  |  |  |  |
| **PA** | 0.443 | 0.590 | 0.521 | 0.394 | **0.507** |  |  |  |
| **SL** | 0.451 | 0.498 | 0.437 | 0.561 | 0.436 | **0.860** |  |  |
| **TDT** | 0.115 | 0.055 | 0.155 | 0.671 | 0.099 | 0.111 | **0.308** | |

Table 5. Hypothesis results

| Hypothesis | Relationship | Coeficiente Path | 𝝆 −valor | Result |
| --- | --- | --- | --- | --- |
| H1 | CM 🡪 SL | 0.103 | 0.141 | Unsupported |
| H2 | CM 🡪 PA | 0.353 | *** | Supported |
| H3 | CPS 🡪 SL | 0.177 | 0.010 | Supported |
| H4 | APS 🡪 SL | 0.083 | 0.207 | Unsupported |
| H5 | TDT 🡪SL | -0.058 | 0.326 | Unsupported |
| H6 | TDT 🡪PA | 0.147 | 0.019 | Supported |
| H7 | ILT 🡪 SL | 0.150 | 0.009 | Supported |
| H8 | ILT 🡪 PA | 0.264 | *** | Supported |
| H9 | CTF 🡪SL | -0.381 | *** | Supported |
| H10 | CTF 🡪 PA | -0.302 | *** | Supported |

**Note.** Own elaboration based on SmartPLS 3.2.7 software. *** Significance less than p < 0.001.

## Supplementary Figures


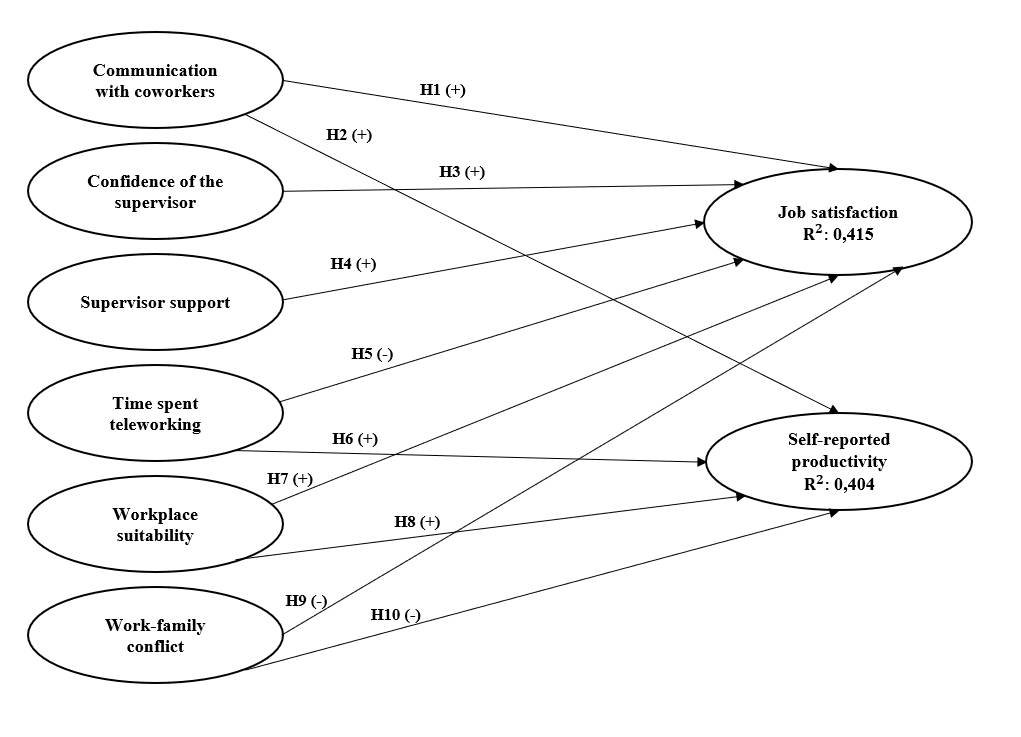


Figure 1. Proposed model.


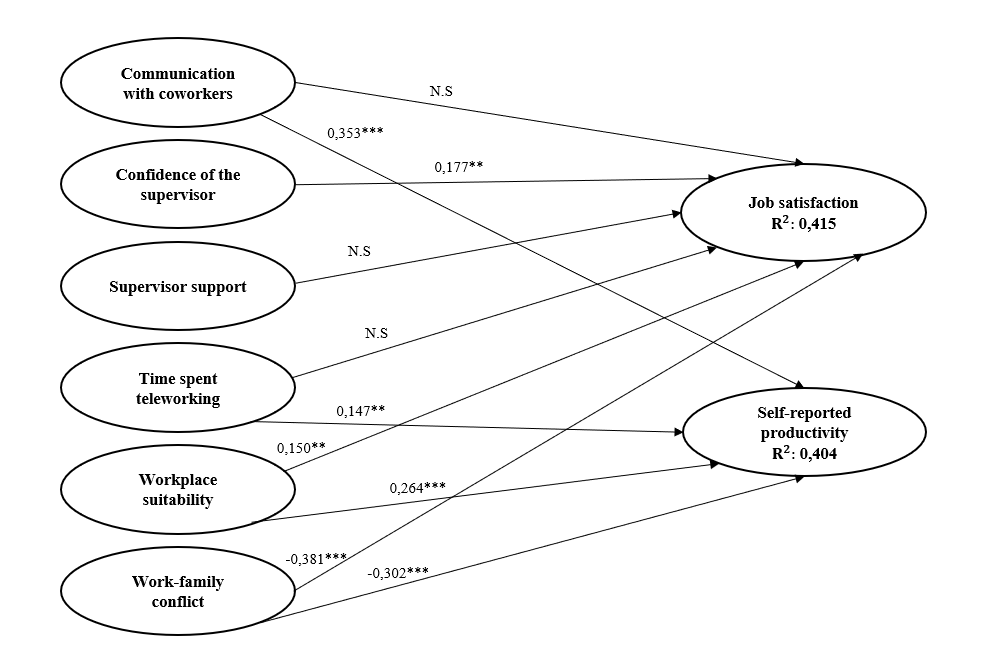


*Figure 2. Structural model.*
